# Septin filament coalignment with microtubules depends on SEPT9_i1 and tubulin polyglutamylation, and is an early feature of acquired cell resistance to paclitaxel

**DOI:** 10.1038/s41419-019-1318-6

**Published:** 2019-01-22

**Authors:** Benjamin Targa, Laurence Klipfel, Isabelle Cantaloube, Joëlle Salameh, Béatrice Benoit, Christian Poüs, Anita Baillet

**Affiliations:** 1 INSERM UMR-S 1193, Univ. Paris-Sud, Université Paris-Saclay, Châtenay-Malabry, France; 2 INSERM UMR-S 968, CNRS UMR 7210, Univ. Pierre et Marie Curie Paris 06, Sorbonne Universités, Paris, France; 3 APHP, Hôpitaux Universitaires Paris-Sud, hôpital Antoine Béclère, Clamart, France

## Abstract

Cancer cell resistance to taxanes is a complex, multifactorial process, which results from the combination of several molecular and cellular changes. In breast cancer cells adapted to long-term paclitaxel treatment, we previously identified a new adaptive mechanism that contributes to resistance and involves high levels of tubulin tyrosination and long-chain polyglutamylation coupled with high levels of septin expression, especially that of SEPT9_i1. This in turn led to higher CLIP-170 and MCAK recruitment to microtubules to enhance microtubule dynamics and therefore counteract the stabilizing effects of taxanes. Here, we explored to which extent this new mechanism alone could trigger taxane resistance. We show that coupling septins (including SEPT9_i1) overexpression together with long-chain tubulin polyglutamylation induce significant paclitaxel resistance in several naive (taxane-sensitive) cell lines and accordingly stimulate the binding of CLIP-170 and MCAK to microtubules. Strikingly, such resistance was paralleled by a systematic relocalization of septin filaments from actin fibers to microtubules. We further show that this relocalization resulted from the overexpression of septins in a context of enhanced tubulin polyglutamylation and reveal that it could also be promoted by an acute treatment with paclitaxel of sensitve cell displaying a high basal level of SEPT9_i1. These findings point out the functional importance and the complex cellular dynamics of septins in the onset of cell resistance to death caused by microtubule-targeting antimitotic drugs of the taxane family.

## Introduction

Paclitaxel induces cell death, making it a successful drug for anticancer chemotherapy. However, several superimposed mechanisms of resistance limit the extent of paclitaxel use in therapeutics^1^. A new mechanism contributing to such chemoresistance was uncovered in the laboratory, involving the overexpression of septins coupled to tubulin modifications^2,3^.

Septins are filamentous GTPases involved in a vast array of cellular functions in which they mainly behave as diffusion barriers or as scaffolds^4,5^. In mammals, there are 13 septin genes grouped in four families^6^. Septins arrange into palindromic octamers: SEPT9-SEPT7-SEPT6-SEPT2-SEPT2-SEPT6-SEPT7-SEPT9, which then can assemble into higher structures like filaments, gauzes or rings^7,8^. Each of the septin gene loci can generate several transcripts. The *sept9* locus engenders at least 15 isoforms^9^ and the overexpression of SEPT9_i1, one of the largest isoforms, has already been involved in ovarian cancer tumorigenesis^10^, head and neck cancers^11^, and breast cancer progression^12,13^. In interphase cells, septins can be found on membranes^14,15^, on actin stress fibers^7,16^ and/or on microtubules (MTs) in a few cell types^17^ where they were proposed to play a role in the regulation of MT guidance and organization^18^.

MT dynamics can be modulated by post-translational modifications (PTMs) of tubulin^19^. The detyrosination/retyrosination cycle^20–22^ was involved in breast cancer cell resistance to paclitaxel^23^. Tubulin polyglutamylation (polyE), which was shown to modulate protein interactions with MTs^24,25^ and thus to control MT dynamics, consists in the branching and in the elongation of (Glu)_n_ side chains on both α- and/or β-tubulin. It is catalyzed by tubulin tyrosine ligase like (TTLL) glutamylases. TTLL4, 5, or 7 start the branching by adding a single glutamate whereas the elongation is catalyzed by TTLL1, 6, 11, or 13^26,27^.

Previous studies have shown that cell resistance to paclitaxel is a multifactorial process^1,28^. In addition, we have shown previously^2,3^ that long-term paclitaxel adaptation of MDA-MB 231 breast cancer cells (paclitaxel-resistant; Tr) resulted in additional changes: (i) in the occurrence of high levels of long-chain polyE and in TTL (Tubulin Tyrosine Ligase)-mediated tubulin retyrosination, (ii) in global septin overexpression together with a partial replacement of SEPT9_i3 (the main isoform of paclitaxel-sensitive cells; Ts) by SEPT9_i1, and (iii) in a higher recruitment to MTs of plus end-tracking proteins (+TIPs) that control catastrophes (MCAK) and rescues (CLIP-170). Knocking-down each of these actors led to the reversion of chemoresistance, allowing us to propose this new resistance mechanism. Strikingly, it was paralleled by a dramatic relocalization of septins from actin filaments to MTs in resistant cells^3^.

Here, to determine to which extent this mechanism alone could trigger taxane resistance, we studied the respective contributions of tubulin modifications and of septin overexpression to the chemoresistant phenotype in MDA-MB 231 taxane-sensitive (Ts) and in a variety of naive cells. We found that the most effective set of modifications consisted in the simultaneous overexpressions of TTLL5, TTLL11, SEPT2, SEPT6, SEPT7 and SEPT9_i1, and that this combination caused the relocalization of septin filaments from actin to MTs, consistent with the phenotype observed in long-term paclitaxel-adapted cells. We also show for the first time that septin relocalization occurred early in response to acute paclitaxel treatment, and that cell lines that constitutively express a high level of SEPT9_i1 were more prone to undergo such a phenotype. Together, these results indicate that septin overexpression and relocalization to MTs is a key event to allow paclitaxel resistance to take place.

## Results

### Paclitaxel resistance is promoted by the overexpression of octamer-forming septins, and is further enhanced by MT polyglutamylation

**Fig. 1:**
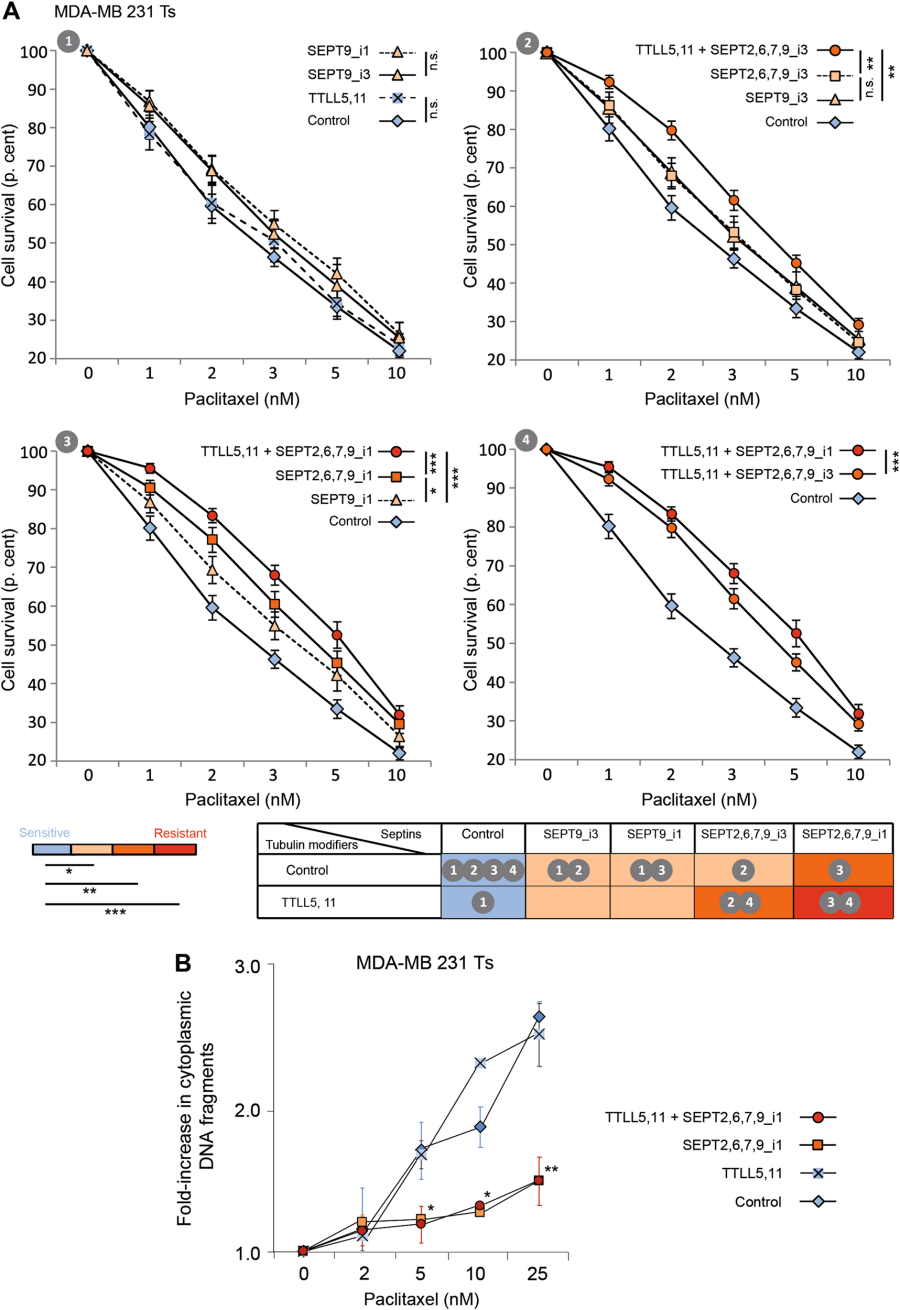
The overexpression of a SEPT9_i1-containing set of filament-forming septins is necessary and sufficient to confer paclitaxel resistance, and is further enhanced upon microtubule polyglutamylation. **a** MTT assays were conducted in paclitaxel-sensitive (Ts) MDA-MB 231 cells in conditions that enhanced either tubulin long-chain polyglutamylation (overexpression of the branching TTLL5 and elongating TTLL11 enzymes) or overexpression of a septin pattern including SEPT9_i1 or_i3, or both. After a 72h-exposure to the paclitaxel concentrations indicated, cell survival was measured. Each point is the mean ± s.e.m of at least 10 independent experiments. Statistical analysis is based on the overall comparison of the curves *p ≤ 0.05, **p ≤ 0.01, ***p ≤ 0.001. Statistical significance of the comparisons with the negative control is color-coded as indicated. The colors also correspond to the additional percentages of cell survival compared to those of control cells at the IC_50_: absence of resistance (blue), low level of resistance (light orange, ≤10%), intermediate level (orange, between 10 and 20%) and high level of resistance (red, >20%). The table summarizes the effect (color-coded) of each effector alone or in combination, and indicates the panel(s) displaying the data. **b** MDA-MB 231 Ts cells were subjected to the overexpression of the indicated plasmids and cell death was quantified 24 h after exposure to paclitaxel by measuring the cytoplasmic release of histone-associated DNA fragments relative to the values without paclitaxel. Each point is the mean ± s.e.m. of at least three independent experiments. *p ≤ 0.05, **p ≤ 0.01

By RNAi depletion, we previously identified four new factors involved in paclitaxel resistance: TTL, TTLLs, septins and +TIPs^3^. Here, in a reverse approach, we investigated which of these actors, alone or in combination, are sufficient to induce a significant level of paclitaxel resistance. As we showed that +TIPs function downstream of tubulin modifications and septins to confer chemoresistance^3^, we focused on the three other factors to determine their role in taxane-resistance acquisition. To this end, we first overexpressed combinations of TTLLs and septins in taxane-senstive (Ts) MDA-MB 231 cells, the cell line from which the long-term paclitaxel-adapted (Tr) cells originated^2^. To determine whether resistance was restricted to breast cancer cells, we also studied cancer cells from other origins (HeLa and HuH7). The study was further extended to immortalized cells (RPE-1) and even to non-human fibroblasts (CHO), to evaluate the universality of the MT-dependent resistance mechanism we uncovered^3^. First of all, transfection efficacy and specificity were verified (Figs. S1 and S2). Drug resistance was determined from experiments that consisted in 72 h-exposure to increasing paclitaxel concentrations followed by measurement of cell survival in MTT assays relative to cells expressing eGFP as a control. Figure 1a.1 shows that the overexpression of TTLL5 (branching) and TTLL11 (elongating) is not sufficient to cause Ts cells to resist paclitaxel. A low level of resistance was induced when each of the SEPT9 isoforms identified in MDA-MB 231 cells (either SEPT9_i3 or the bad prognosis SEPT9_i1 isoform) was overexpressed alone (Fig. 1a.1). When the whole set of filament-forming septins (SEPT2,6,7,9) was overexpressed, especially when it included SEPT9_i1 (Fig. 1a.3) or when MT polyglutamylation was enhanced in the case of the presence of SEPT9_i3 (Fig. 1a.2), a proportion of Ts cells resisted paclitaxel up to ~10 nM. The overexpression of the SEPT9_i1 isoform together with that of other septins enhanced cell resistance to paclitaxel to an intermediate level, and resistance was even higher when septin overexpression was combined with that of polyglutamylation enzymes (Fig. 1a.3 and 1a.4). To reinforce the significance of the above MTT assays, we further verified that the overexpression of septins in association or not with polyglutamylases limits the release of DNA fragments in the cytoplasm, reflecting the reduction in apoptotic cell death (Fig. 1b).

Overexpressing TTL alone also caused cells to resist paclitaxel to an intermediate level (Fig. 2.1). Note that combining TTL overexpression with that of septins alone, whatever the SEPT9 isoform (Fig. 2.1), or with that of the polyglutamylases TTLL5 and 11 did not further enhance Ts cells resistance (Fig. 2.2 and 2.3). When SEPT9_i1 was included in the combination, overexpressing TTL on top of TTLLs even had a negative effect on resistance (Fig. 2.3). In our model of taxane resistance, TTL was an important upstream actor to allow effective long-chain polyglutamyation of tubulin and septin recruitment to MTs^3^. The fact that TTL overexpression failed to increase the resistance conveyed by TTLL and septin overexpression revealed that overexpressed TTLLs bypassed the need for TTL to stimulate long-chain polyE. It also indicated that TTL conferred some level of taxane resistance independently from the polyE/septins pathway, in keeping with the notion that tubulin tyrosination is required for the recruitment of CLIP-170 and MCAK on MTs^29,30^.

**Fig. 2:**
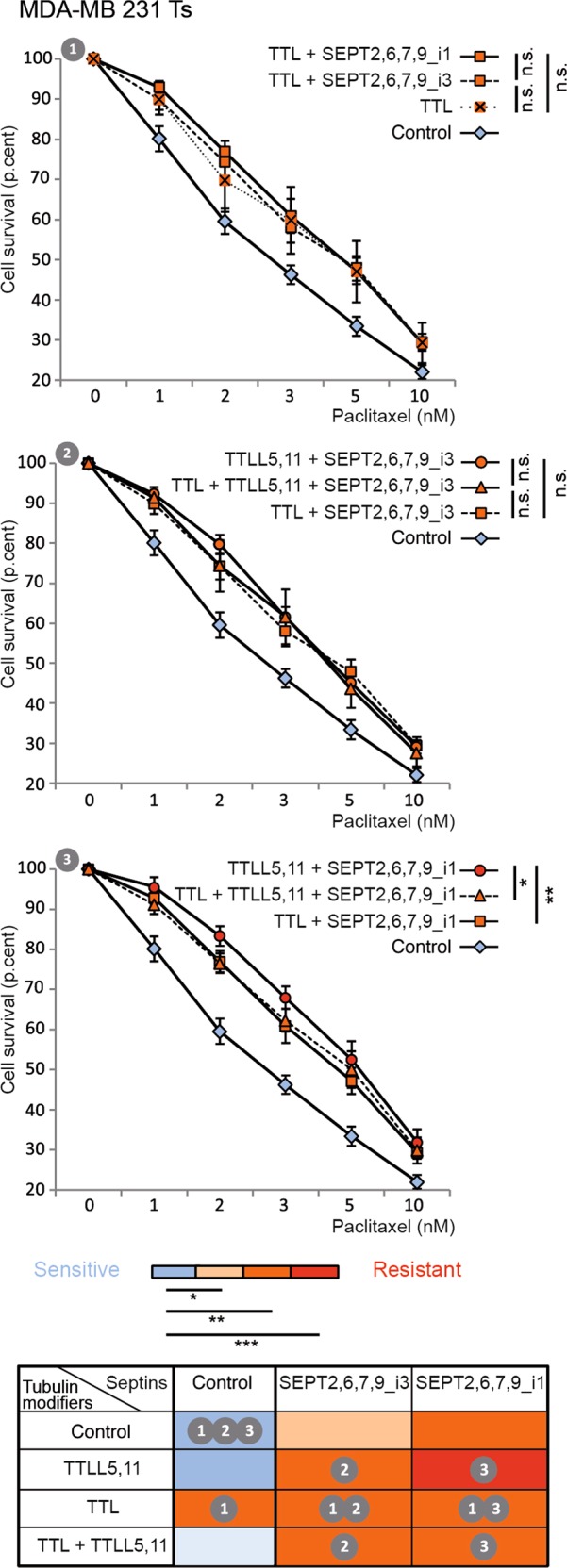
TTL overexpression does not enhance paclitaxel resistance induced by the overexpression of septins and polyglutamylases. MTT assays were conducted in paclitaxel-sensitive (Ts) MDA-MB 231cells in conditions that enhanced or not tubulin retyrosination (overexpression of the Tubulin Tyrosine Ligase TTL) combined with increased tubulin long-chain polyglutamylation (overexpression of TTLL5 and TTLL11 enzymes) or overexpression of a septin set including SEPT9_i1 or_i3, or both. Each bar is the mean ± s.e.m of at least 10 independent experiments. Statistical analysis is based on the overall comparison of the curves *p ≤ 0.05, **p ≤ 0.01, ***p ≤ 0.001. Statistical significance of the comparisons with the negative control is color-coded as indicated. The colors also correspond to the additional percentages of cell survival compared to those of control cells at the IC_50_: absence of resistance (blue), low level of resistance (light orange, ≤10%), intermediate level (orange, between 10 and 20%) and high level of resistance (red, >20%). The table summarizes the effect (color-coded) of each effector alone or in combination, and indicates the panel(s) displaying the data

**Fig. 3:**
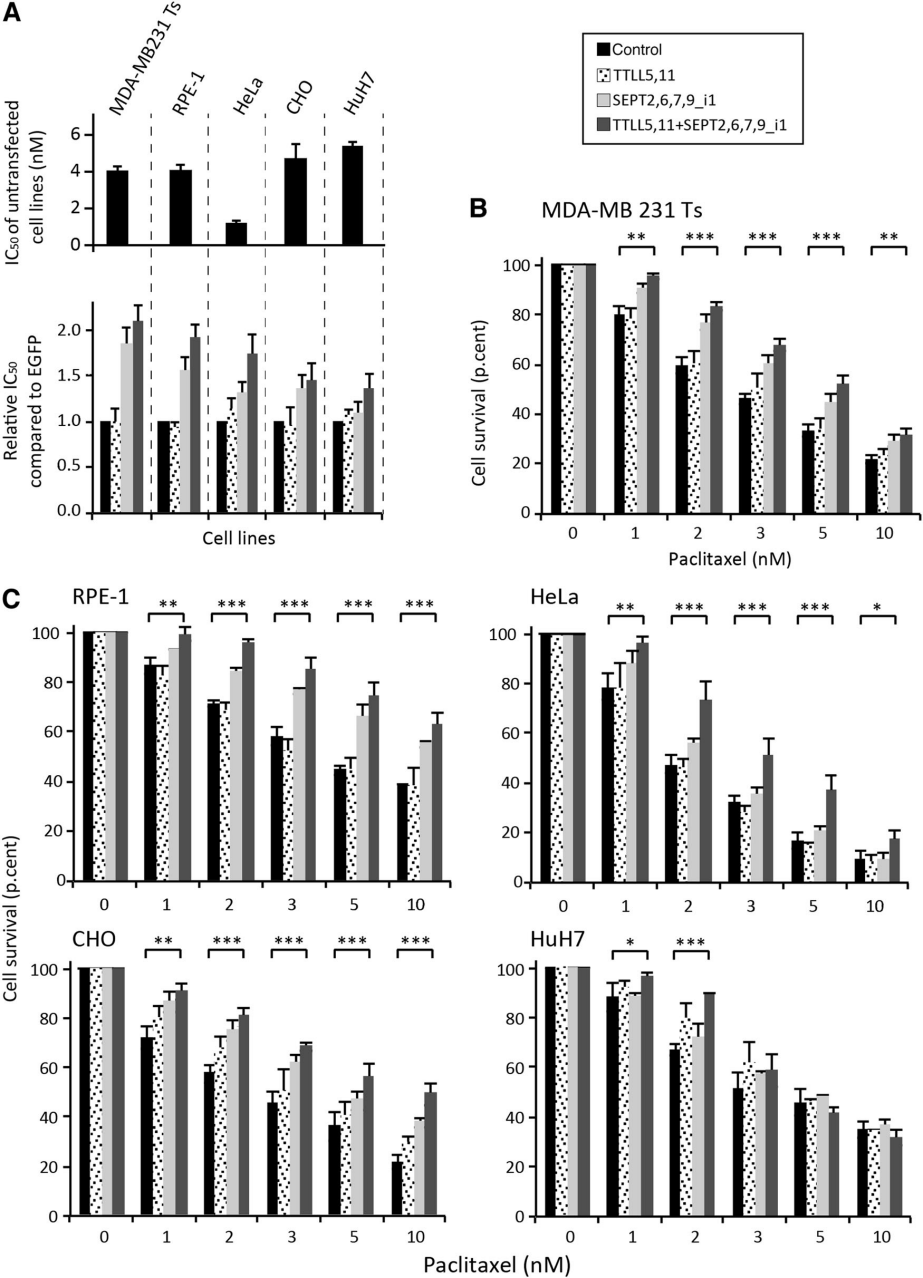
Long-chain polyglutamylation of tubulin and septin overexpression are necessary and sufficient to confer paclitaxel resistance to multiple cell lines. **a** The IC_50_ for paclitaxel of untreated paclitaxel-sensitive (Ts) MDA-MB-231cells, RPE-1, HeLa, CHO and HuH7 cells or in conditions that enhanced either tubulin long-chain polyglutamylation or septin pattern overexpression including SEPT9_i1 or both, were measured using MTT assays with drug concentrations ranging from 0 to 100 nM paclitaxel. The values shown are the mean ± s.e.m. of at least four independent experiments. **b**, **c** MTT assays were conducted in paclitaxel-sensitive (Ts) MDA-MB-231cells, RPE-1, HeLa, CHO, and HuH7 cells in conditions that enhanced either tubulin long-chain polyglutamylation or septin pattern overexpression including SEPT9_i1, or both. After a 72h-exposure to the paclitaxel concentrations indicated, cell survival was measured. Each bar is the mean ± s.e.m of at least four independent experiments. *p ≤ 0.05, **p ≤ 0.01, ***p ≤ 0.001

The same viability assays were repeated in RPE-1, HeLa, CHO and HuH7 cells, which are all sensitive to paclitaxel as evidenced by their respective IC_50_ (Fig. 3a, top panel). MTT cell survival assays were conducted in four different conditions according to the levels of septin expression and of tubulin polyglutamylation: Control, TTLL5,11, SEPT2,6,7,9_i1 and TTLL5,11 + SEPT2,6,7,9_i1. Except for HuH7 hepatoma cells, which displayed low to intermediate resistance only at very low concentrations of paclitaxel (1 nM and 2 nM, respectively), each of the other cell lines behaved like MDA-MB 231 Ts cells (Figs. 3b, c and S3). They resisted paclitaxel when the whole set of polyglutamylation enzymes and septins was overexpressed. The overexpression of septins alone caused a milder resistant phenotype. Regarding polyglutamylase overexpression, it consistently failed to cause resistance, except perhaps in CHO and HuH7 cells at 2 nM paclitaxel (Fig. 3c). Figure 3a (bottom panel) shows the relative increases in IC_50_ for paclitaxel, as determined from the responses to MTT cell survival assays (Fig. 3b, c).

### Septins and tubulin polyglutamylation contribute to CLIP-170 and MCAK recruitment to MTs

**Fig. 4:**
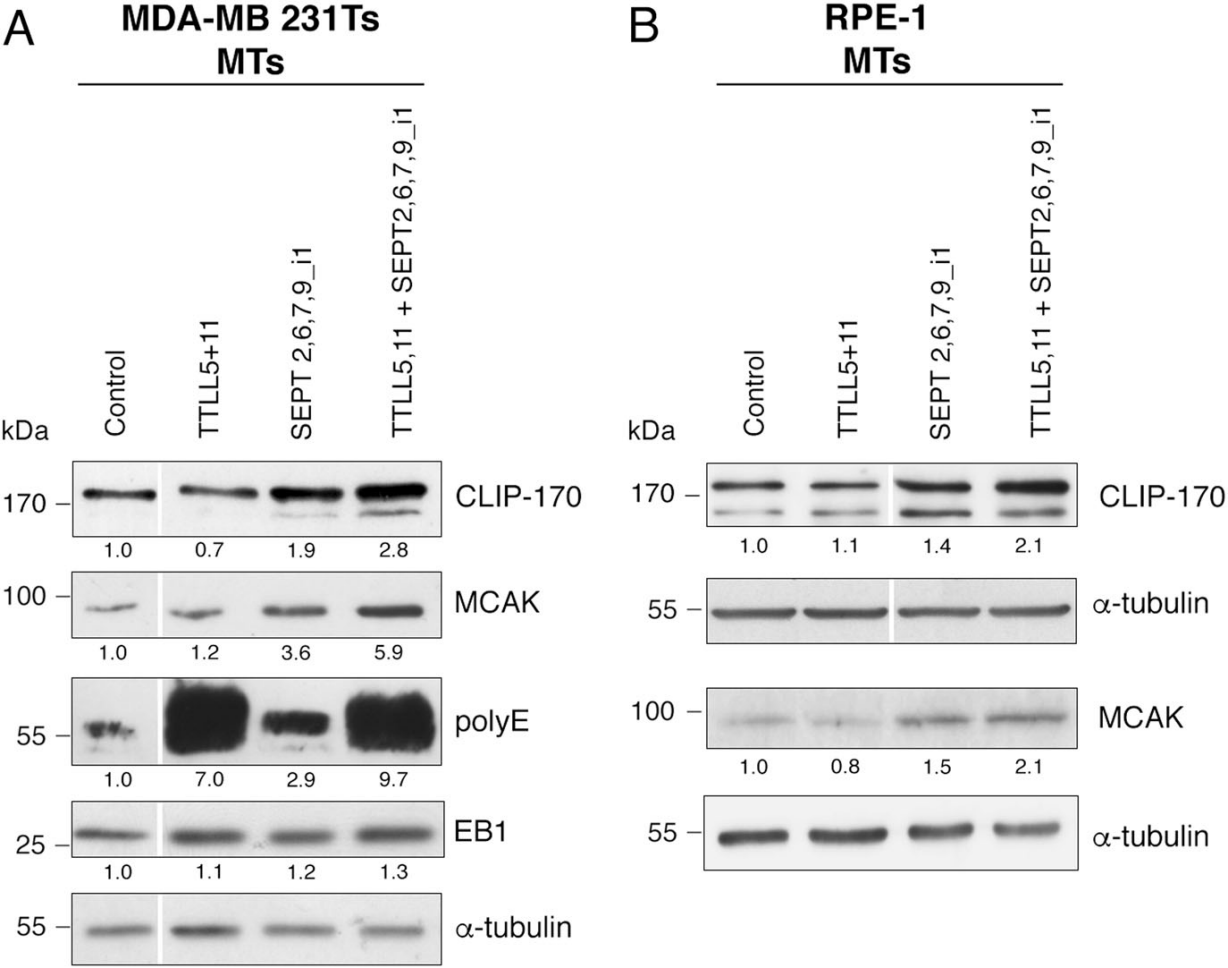
Overexpression of a SEPT9_i1-containing set of septins stimulates the recruitment of CLIP-170 and MCAK to microtubules, which is amplified by long-chain tubulin polyglutamylation. MT fractions from Ts (**a**) or RPE-1 (**b**) cells subjected to TTLL and/or septin overexpression as indicated were analyzed for the recruitment of various +TIPs (EB1, CLIP-170, and MCAK). The polyE antibody recognizes glutamate chains composed of at least three glutamic acids. The polyE signal is therefore a reflection of elongating TTLL activity. Tubulin was used as a loading control. The quantitative data are the ratio of the intensity of the protein of interest to that of α-tubulin for the blot shown

We previously showed in our Tr model^3^ that the increase of CLIP-170 and MCAK on MTs contributed to chemoresistance, downstream of tubulin modifications and septin recruitement to MTs. As tubulin tyrosination is a key factor to allow the binding of these +TIPs to MTs^29,30^, we next wondered whether tubulin polyglutamylation and/or septins also contributed to this recruitment. In a way that paralleled what we observed above for the induction of paclitaxel resistance, Ts cells overexpressing the combination of TTLL5,11 alone did not display a higher recruitment of CLIP-170 and MCAK in MT fractions (Fig. 4a). In contrast, overexpressing a combination of septins (SEPT2,6,7,9_i1) increased CLIP-170 and MCAK levels in the MT fractions. This effect was even higher when long-chain tubulin polyglutamylation was stimulated in parallel (Fig. 4a). Note that these phenotypes occurred while TTL was not overexpressed, suggesting that an increased recruitment of these +TIPs is also stimulated by septin recruitment on MTs. Increased levels of CLIP-170 and MCAK in the MT fractions were also observed in the RPE-1 cell line (Fig. 4b), confirming that the overexpression of septins combined with that of polyglutamylases may cause an enhanced recruitment of these proteins not only in cancer cells.

### The combined overexpression of septins and of polyglutamylation enzymes that optimizes paclitaxel resistance causes the relocalization of septin filaments to MTs, independently of paclitaxel stimulation

**Fig. 5:**
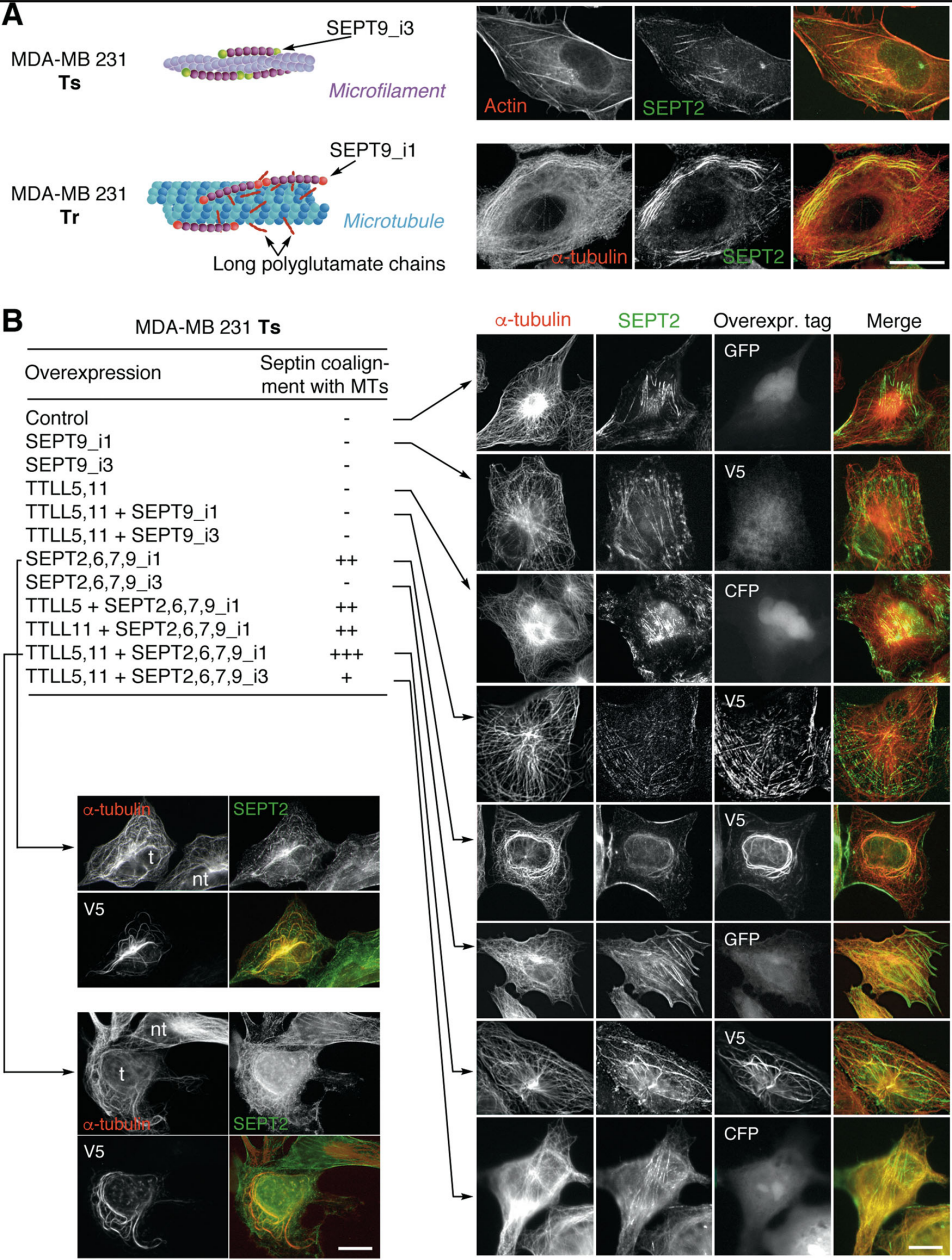
Paclitaxel-independent septin relocalization from actin fibers to microtubules is complete upon the overexpression of SEPT9_i1-containing set of septins combined with long-chain polyglutamylation in the MDA-MB 231 breast cancer cell line. **a** Subcellular localization of septin filaments in the paclitaxel-sensitive (Ts) and -resistant (Tr) MDA-MB 231 cell lines. Septin filaments associated with actin fibers in Ts cells mainly comprise SEPT9_i3, while SEPT9_i1 is the main long SEPT9 isoform recovered in septin filaments, which are associated with long-chain polyglutamylated MTs in paclitaxel-adapted Tr cells^3^. Endogenous SEPT2 was used to reveal the localization of septin filaments in Ts and Tr cells. **b** Subcellular septin localization in Ts cells overexpressing various combinations of septins and TTLL enzymes alone or in association. SEPT2 labelling was used to detect endogenous septin filaments. With the exception of SEPT9_i1, which bears the V5 tag, all septins are GFP-tagged. TTLL enzymes are coexpressed with CFP. The bottom-left panels show examples of differential septin localization in transfected (t) versus non-transfected (nt) cells in the same field. The images shown are representative of at least three independent experiments. Scale bars = 10 µm

Another striking phenotype that distinguished Ts from Tr cells is the subcellular localization of septin filaments that shifted from actin fibers to MTs, respectively (ref. ^3^ and Fig. 5a). Furthermore, SEPT9_i1 expression was increased in Tr cells while SEPT9_i3 was the main SEPT9 isoform in Ts cells. Also, SEPT9_i1-enriched septin filaments are associated with MTs that bear long polyglutamate side chains in resistant Tr cells^3^. We wanted to determine to which extent this change of localization resulted from the induction of tubulin polyglutamylation and/or from the expression of a specific SEPT9 isotype.

We thus tested which of tubulin long-chain polyglutamylation and/or septin overexpression (including SEPT9 isoform variation) was required to make the endogenous septin filaments coalign with MTs. As shown in Fig. 5b, the stimulation of tubulin polyglutamylation alone was not sufficient to displace septin filaments from actin fibers to MTs. Next, all the conditions in which SEPT9 alone (either the _i1 or the _i3 isoform) was overexpressed, in combination or not with the polyglutamylation enzymes, also failed to relocalize endogenous septin filaments, indicating that SEPT9 (even the _i1 isoform which can physically interact with tubulin^31^) does not have a role by itself in this change of septin subcellular localization. Moreover, as the overexpression of an individual septin may form artifactual homo-oligomers^32^ and mimic physiological filament coalignment with MTs, we also overexpressed SEPT9 isoforms together with other filament-forming septins and followed total SEPT2 (endogenous and overexpressed) by immunofluorescence to actually reflect the behavior of endogenous septin filaments. In these conditions, when filament-forming septins included SEPT9_i1, a partial relocalization of septin filaments to MTs could be observed, mostly on perinuclear MTs forming bundles (Fig. 5b). This relocalization was complete when septin overexpression was combined with effective long-chain polyglutamylation that resulted from the overexpression of both TTLL5 and TTLL11 (Fig. 5b). The latter situations contrasted with the conditions that comprised SEPT9_i3. In these cases, overexpressing septins alone did not allow septin filaments to relocalize from actin fibers to MTs (Fig. 5b), and combining the overexpression of this septin profile with long-chain polyglutamylation only moderately caused septin filaments to coalign with MTs, which is also in accordance with the lower level of resistance shown by cell viability data (see Fig. 1, right panels).

**Fig. 6:**
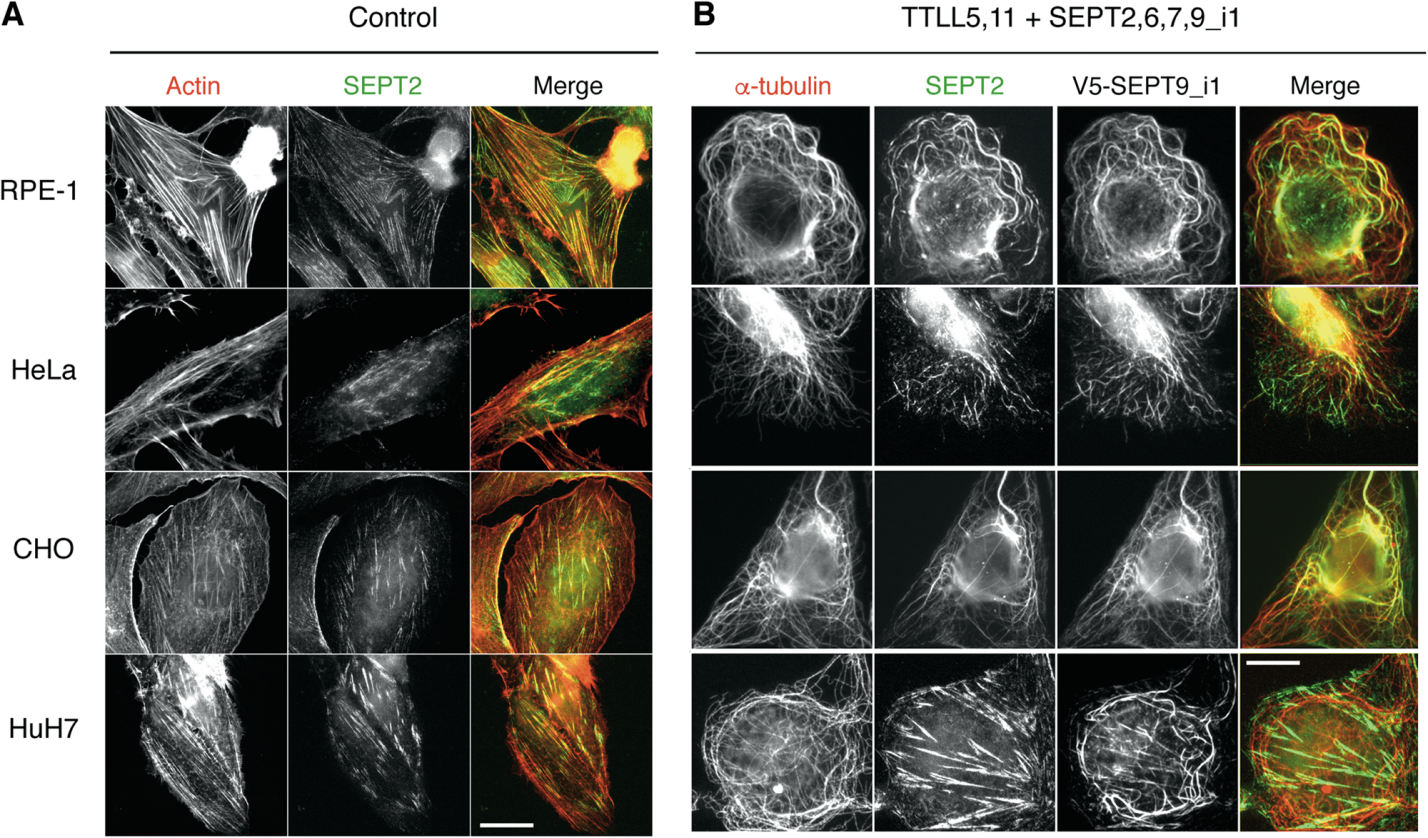
Paclitaxel-independent subcellular relocalization of endogenous septin filaments to microtubules occurs following the combined overexpression of polyglutamylases (TTLL5,11) and septins (2,6,7,9_i1) in diverse cell lines. **a** Immunofluorescent images of endogenous SEPT2 (green) and actin (phalloidin; red) in cells. The overlay images show that septin fibers colocalize with actin stress fibers in each cell line. **b** Immunofluorescent images show endogenous SEPT2 (green), overexpressed SEPT9_i1 and alpha-tubulin (red). Septin filaments relocalize to MTs when polyglutamylases and septins (including SEPT9_i1) are overexpressed in CHO, RPE-1, and HeLa cells and only partially in HuH7 cell line. The images shown are representative of at least three independent experiments. Scale bars = 10 µm

The functional importance of combining long-chain tubulin polyglutamylation and SEPT9_i1 expression to trigger septin coalignment with MTs was further verified in other cell types. As shown in Fig. 6, the relocalization of endogenous septin filaments was also observed in RPE-1, HeLa and CHO cells, the same 3 cell lines in which paclitaxel resistance could be significantly induced (see Fig. 3c). Consistent with this parallel between paclitaxel resistance and septin relocalization, HuH7 cells, which only resisted very low concentrations of paclitaxel (see Fig. 3c), did not exhibit a displacement of their septin filaments to MTs, as visualized by the labelling of SEPT2 (Fig. 6, bottom line).

### Cells that survive acute paclitaxel treatment exhibit MT-bound septins

**Fig. 7:**
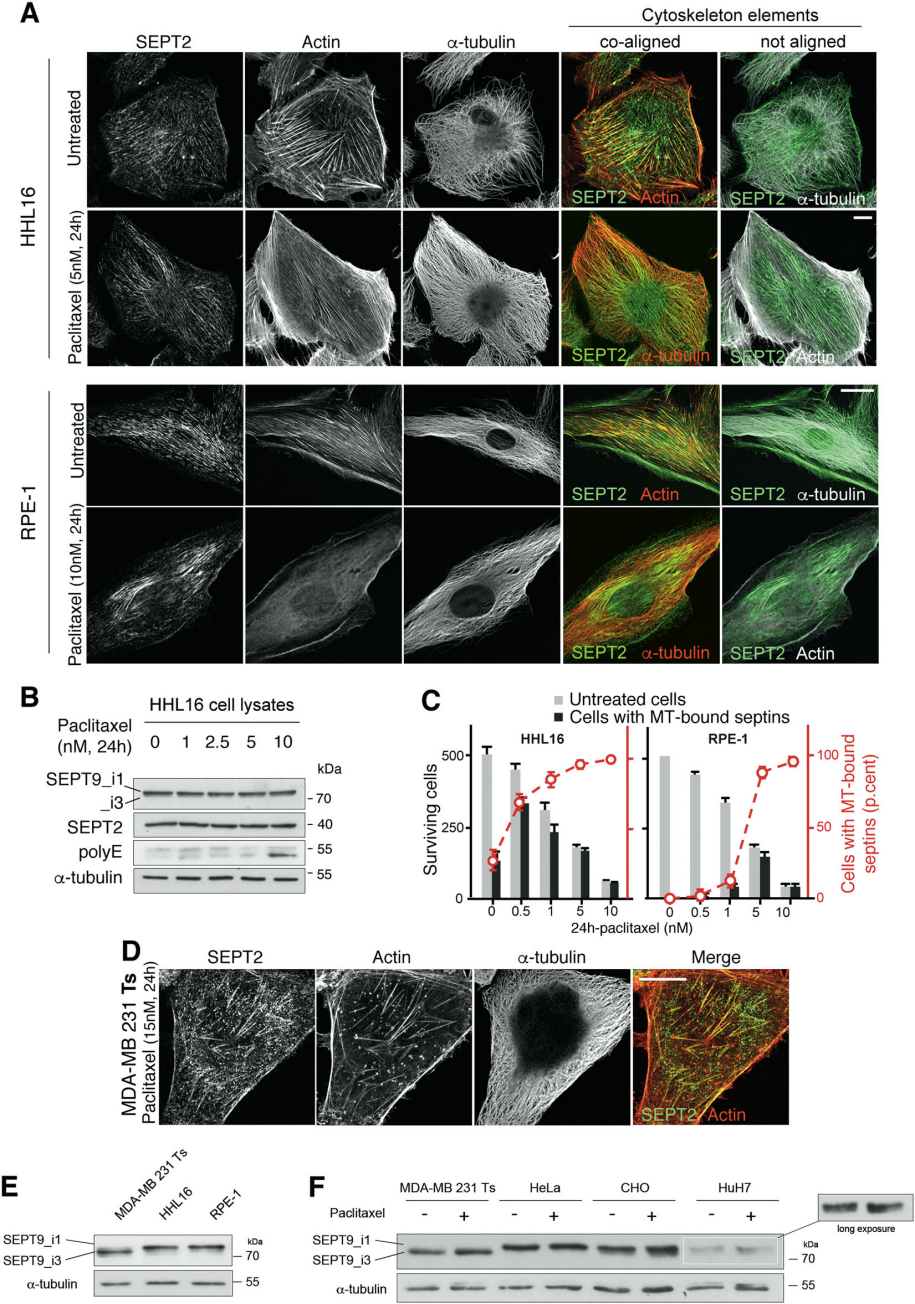
Cells that survived acute paclitaxel treatment exhibit subcellular relocalization of endogenous septin filaments to microtubules. **a** Immunofluorescent images of endogenous SEPT2 (green), actin (phalloidin; red or white) and α-tubulin in HHL16 and RPE-1 cells following 24 h paclitaxel treatment. The overlay images show that septin coalign with actin fibers in untreated cells, and with tubulin in paclitaxel treated cells. The paclitaxel concentration used corresponds to three times the IC_50_ for the drug. **b** Septin cellular amount does not change upon treatment with increasing concentrations of paclitaxel. Note that tubulin polyglutamylation starts increasing only after 24 h of 10 nM paclitaxel treatment. **c** Quantification of cells with MT-bound septins among cells that have survived 24 h-paclitaxel treatment. **d** Immunofluorescent images of breast cancer MDA-MB 231 cells after 15 nM paclitaxel treatment for 24 h. Note that septin filaments are still mainly associated with actin fibers in these cells. **e** Immunoblot showing the basal levels of SEPT9 long isoforms (SEPT9_i1 and _i3) in total cell extracts of untreated sensitive MDA-MB 231 Ts, HHL16, and RPE-1 cells. **f** Immunoblot showing the basal levels of SEPT9_i1 and _i3 in total cell lysates of untreated or paclitaxel-treated MDA-MB 231 Ts, HeLa, CHO, and HuH7 cells. The images, immunoblots shown and cell quantifications are representative of at least three independent experiments. Scale bars = 10 µm

Our above results show that septin relocalization triggered by septin overexpression (including SEPT9_i1) in a context of long-chain tubulin polyglutamylation was well correlated with cell survival to a 72 h exposure to paclitaxel. To confirm that this change in septin subcellular location could be actually causative of the onset of a cell resistance, we treated different untransfected cell lines using 24 h acute treatment with paclitaxel at a concentration three-times higher than their respective IC_50_. Figures 7a, d, S4 and S5 show that, except for MDA-MB 231 Ts, a partial or complete relocalization of septin filaments occurred from the actin cytoskeleton to the MT network. As this relocalization seems to be a general feature of cancer and non-cancer cell response to acute paclitaxel treatment, we further analyzed this cytoskeleton reorganization in the two RPE-1 and HHL16 immortalized cell lines that were both good responders to paclitaxel and easy to image. We next eliminated the hypothesis that the change in septin location could originate from enhanced septin expression following paclitaxel treatment (Fig. 7b). It is worth noting, especially in RPE1 (Fig. 7a) or HeLa (Fig. S5) cells, that the relocalization of septins to MTs is accompanied by a loss of thick actin stress fibers. This is in agreement with the role of septins in stabilizing actin fibers^33^. We also noticed that the higher the concentration of paclitaxel we used, the faster septins relocalized to MTs (Data not shown). Measuring the time-course of cell survival in both RPE-1 and HHL16 cell lines showed that strikingly, all the cells that survived the two highest concentrations of paclitaxel, exhibited septin filaments coaligned with their MTs (Fig. 7c), further indicating that when it actually occurs, this septin relocalization plays a key role in cell survival/resistance to paclitaxel treatment. As long-term adapted MDA-MB 231 Tr cells exhibit an elevated level of SEPT9_i1 compared to their sensitive counterpart Ts^3^, the differential behavior between MDA-MB 231 Ts, HuH7 and the other cell lines is likely to originate from differences in the basal level of SEPT9_i1. Indeed, all the cell lines that displayed strong septin relocalization after paclitaxel treatment actually exhibited a high basal level of SEPT9_i1 expression (Fig. 7e, f). The low basal level of SEPT9_i1 expression in HuH7 and its absence in MDA-MB 231 Ts cells correlates well with their respective partial or barely detectable relocalization of septin filaments to MTs (Fig. 7f). In addition, short-term paclitaxel treatment failed to stimulate SEPT9_i1 expression in MDA-MB 231 Ts and HuH7 cells, pointing out that a high basal level is determinant to induce such a quick relocalization, whether the cells are cancerous or not. This result also suggests that cell lines with a low basal level of SEPT9_i1 expression might need a longer adaptation to resist the drug.

## Discussion

Cancer cell resistance to taxanes is a multifactorial process that involves a great variety of cellular adaptation means^1,28^. We previously uncovered a new septin- and tubulin polyglutamylation-dependent mechanism that contributes to long-term cell adaptation to taxanes^3^. While the inhibition of each of these actors partially restored paclitaxel sensitivity^3^, here we sought to determine to which extent they were sufficient to trigger resistance in various paclitaxel-naive cells. We show that modifying tubulin alone failed to cause resistance. In contrast, overexpressing a set of filament-forming septins (SEPT2,6,7,9), especially when it comprised SEPT9_i1, was sufficient to cause significant paclitaxel resistance that was further enhanced upon the induction of MT polyglutamylation. Strikingly, this resistance appeared to be tightly linked to the early relocalization of septin filaments from actin fibers to MTs, as shown upon acute paclitaxel treatment.

Tubulin polyglutamylation is a complex modification^34^, which plays an important role in neuropathology as shown in mice defective for a glutamate chain-reducing enzyme (CCP1), which display Purkinje cell degeneration^35,36^. A link between tubulin polyglutamylation and cancer cell resistance to MT-targeting agents has already been proposed in estramustine-resistant prostatic cancer cells^37^, but no mechanism was proposed to explain it.

Septin filaments, which have been identified as a new cytoskeleton element^38^, may display a great variety of subcellular organizations according to the organelle or structure they associate with. Apart from their assembly on membranes of specific curvature and lipid composition^39^ or their ability to form free rings in the cytoplasm^7^, they also associate with cytoskeleton components, mostly with actin filaments^16,40,41^ and with MTs in a few cell types^7,17,42,43^. The control of septin subcellular localization may thus regulate key cellular functions^44,45^. When septins are associated with actin microfilaments, they may impact on cell morphology, actin cytoskeleton organization, and cell migration^46–48^. They would function by crosslinking actin microfilaments in stress fibers, protecting them against depolymerization^33^. In agreement with this role, we observed that upon acute paclitaxel treatment, thick actin stress fibers disappear when septin filaments relocalized to MTs (see Fig. 7a). When coaligned with MTs, septins contribute to promote cell asymmetry^49^. Indeed, in polarized epithelial cells, post-Golgi trafficking operates along polyglutamylated MT tracks that bear septins^50^. Septins can also enhance MT dynamics by preventing the binding of the stabilizing protein MAP4 to MTs^51^ and/or by enhancing the recruitment of the rescue-promoting and catastrophe-promoting factors CLIP-170 and MCAK, respectively (ref^3^. and this study). We further show now that septin relocalization to MTs is linked to the early development of paclitaxel resistance.

Septin expression is modulated in pathology including in cancer^4,6,52^. Some septins like SEPT2, SEPT8, SEPT9, and SEPT11 are often overexpressed while SEPT4 and SEPT10 are frequently downregulated^5,53^. Many cancers also display differences in the level of SEPT9 isoform expression^54^. SEPT9_i2, SEPT9_i3, and SEPT9_i4 are often downregulated^13,55^ while SEPT9_i1 and SEPT9_i5 are overexpressed in various human tumors^10–13,56^. In our experiments, we observed a perfect correlation between the efficacy of early relocalization of septin filaments to MTs and the basal level of SEPT9_i1 expression (Fig. 7a–f and Fig. S5). SEPT9_i1 has already been proposed to be a marker of bad prognosis^11,57^ and to correlate with cell resistance to MT-disrupting agents^58^. The mechanisms proposed thus far include a role for SEPT9_i1 in the control of HIF-1α nuclear translocation and transcriptional effects^59,60^ or in a putative stimulation of MT dynamics through a delay in JNK degradation^61,62^. The latter effect could also contribute to cell adaptation to the MT-stabilizing action of taxanes. In addition, the SEPT9_i1-facilitated relocalization of septin filaments to MTs (this manuscript) could represent another means of modulating MT dynamics as recently shown in vitro^63^ and as observed in our long-term adapted cells^3^. Even though all cell types may adapt to taxanes following long-term exposure to paclitaxel as exemplified in our Tr cells^2,3^, the basal level of SEPT9_i1 could thus be predictive of the easier emergence of cell resistance to taxanes, especially upon acute treatments. Moreover, the fact that a broad range of cells express a sufficient level of SEPT9_i1 suggests that this mechanism could be relevant not only in cancer cells but also in any other tumor-associated cells.

Some molecular determinants of septin association with cytoskeleton elements have been identified, but the reasons for which septins associate with either cytoskeleton element are still far from clear. It has been shown that septins may bind directly to actin filaments^64^ or to actin-associated proteins like non-muscular myosin II^41^ or anillin^7^. SEPT9 can directly interact with F-actin^33^ via its N-terminal region. The same region is also involved in the interaction with tubulin through basic motifs present in the long isoforms of SEPT9 (SEPT9_i1, _i2 and _i3)^31^. SEPT9_i1 but not SEPT9_i3, when overexpressed with other octamer-forming septins, can specifically contribute to endogenous septin filament binding to MTs, and especially when the latter are polyglutamylated (these data). In spite of the minor sequence differences between the three long SEPT9 isoforms, SEPT9_i2 was recently shown to limit tumorogenicity contrary to SEPT9_i1^55^. SEPT9_i1 only differs from SEPT9_i3 by the presence of an additional 17-aminoacid sequence at its N-terminus and by its next eight aminoacids, which makes SEPT9_i1 to bear five more positive charges than SEPT9_i3. Such difference would favor SEPT9_i1 over the other long isoforms for interacting with the acidic tail of tubulin, and even more when tubulin bears long polyglutamylate chains. Such interaction could be a cause of septin filament retention on the MTs of a variety of cells, making them develop a resistance to low concentrations of paclitaxel.

However, it is likely that other proteins are involved in stimulating septin interaction with MTs. Indeed, SEPT9_i1 is necessary but not sufficient to relocalize septin filaments to MTs, as high basal levels of SEPT9_i1 alone do not cause septins to coalign with MTs, as illustrated in RPE-1 or HHL16 cells (Fig. 7). Cell exposure to paclitaxel is also needed to trigger this relocalization. The molecular determinants of septin compartmentalization may involve a complex interplay between MTs, septin filaments including SEPT9_i1, and actin filaments, which will deserve further exploration, with a special emphasis on Borg proteins, which were evidenced to reinforce the binding between septin and actin filaments in non-dividing cells^65^. Some motors may also be involved as the EB1-binding kinesin KIF17 has been shown to bind SEPT9^66,67^. Targeting this re-localization process would be a valuable approach to limit cell adaptation and resistance to cancer chemotherapies using MT poisons in the future.

## Materials and methods

### Cell culture and treatments

Experiments were performed with human cancer cells: MDA-MB 231 (breast), HeLa (cervix), HuH7 (hepatoma), human immortalized cells: RPE-1 (retinal epithelium), HHL16 (hepatocytes), or Chinese Hamster Ovary fibroblasts (CHO). All cell lines were cultured with 5% CO_2_ in Dulbecco’s Modified Eagle Medium (DMEM, Gibco™, Fisher Scientific, Illkirch, France) containing an antibiotic–antifungal mixture and supplemented with 10% fetal calf serum (GE Healthcare HyClone™, Fisher Scientific), 1% sodium pyruvate and 1% L-Glutamine for all the cell lines except MDA-MB 231 (2%). The paclitaxel-resistant (Tr) and -sensitive (Ts) sublines of the human breast carcinoma cell line MDA-MB 231 were continuously cultured without (Ts) or with 25 nM paclitaxel (Tr) as previously described^2^. When appropriate, cells were treated with different concentrations of paclitaxel (Sigma, Darmstadt, Germany) for 24, 48 or 72 h.

### Cell transfections

Transfections of RPE-1 and HuH7 cells were performed 48 h or 72 h prior to analysis using X-tremeGENE while those of Ts, HeLa, and CHO cells were done with Turbofect (ThermoFischer, Illkirch, France), according to the manufacturer’s instructions. Note that the HHL16 cell line was not used in these experiments because they are hard to transfect. Plasmids were used alone or up to a combination of six but the maximal amount of total DNA used per well (6-well plate, 50% confluence) never exceeded 1.5 µg. The amounts of plasmids were determined from the measurements of the efficacy of transfection of the combination involving the six plasmids of interest (SEPT2, SEPT6, SEPT7, SEPT9_i1 or SEPT9_i3, TTLL5, and TTLL11; Fig. S1). Importantly, the same individual amounts were used in every transfection experiment even when the plasmids were used alone. The pEGFP plasmid (Addgene) was used as a negative control. GFP-TTL, CFP-TTLL5, and CFP-TTLL11 expression vectors were kind gifts of Dr. C. Janke (Institut Curie, Orsay, France). GFP- and His-tagged septins (SEPT2, 6, 7 or 9_i3) and V5-SEPT9_i1 were kindly provided by Pr W. Trimble (Hospital for Sick Children, Toronto, Canada) and Dr. A. Gassama (CHB Paul Brousse, Villejuif, France), respectively.

### Cell viability assays

Twenty-four hours after cell transfection or not, sensitivity to paclitaxel was measured using either 3-(4,5-Dimethylthiazol-2-yl)-2,5-diphenyltetrazolium bromide (MTT, Sigma) cytotoxicity assay after 72 h exposition to increasing concentrations of paclitaxel, or the Cell Death Detection ELISA^PLUS^ assay, according to the manufacturer’s instructions (Roche, Sigma) after 24 h paclitaxel treatment. The IC_50_ was determined with Prism4 software (GraphPad software). Each value represents the mean ± s.e.m. of at least four independent experiments.

### Immunoblotting

Total cell lysates and MT fractions were prepared as previously described^2^. Constant protein amounts (10 μg) were loaded onto gels for total cell lysate analysis, while constant α-tubulin amounts were used for MT fraction analysis. Blots were probed with selected primary antibodies against: α-tubulin (clone DM1-A, Sigma), SEPT2 (Atlas, Ozyme, St Quentin en Yvelines, France), SEPT9 (Proteintech, Manchester, United Kingdom), EB1 (H70, Santa Cruz Biotechnology, Clinisciences, Nanterre, france), CLIP-170 (H300, Santa Cruz Biotechnology), MCAK (Abnova, VWR, Fontenay sous bois, France), GFP (Cell Signaling, Ozyme), V5 (Invitrogen, ThermoFischer). The anti-long chain-polyglutamylated-tubulin (polyE) antibody was prepared and characterized by Drs C. Janke (Institut Curie, Orsay, France) and T. Surrey (London Research Institute, London, England). Protein bands were visualized with respective HRP-conjugated secondary antibodies and the ECL detection kit (Pierce, ThermoFischer). Western-blot quantification was performed after film digitization using the ImageJ software (National Institutes of Health, Bethesda, MD; imagej.nih.gov/ij/).

### Immunofluorescence and microscopy

Cells grown on glass coverslips were immunostained with primary α-tubulin (clone DM1-A, Sigma), SEPT2 (Atlas), GFP (Cell Signaling) or V5 (Invitrogen) antibodies, after fixation with 3.75% paraformaldehyde/PBS, permeabilization with 0.1% Triton X-100/PBS and blocking with 0.5% BSA/PBS. Secondary antibodies were Alexa Fluor 488- or 555-conjugated antibodies against rabbit or mouse IgGs (Molecular Probes, Invitrogen) and Cyanine 5 antibody against mouse IgGs (Abcam, Paris, France). Actin microfilaments were visualized by fluorescent phalloidin (Sigma).

Images were acquired using either a Zeiss LSM 510 confocal microscope (63 × 1.4 NA objective) or a Leica DMLB microscope (40 or 100 × 1.3 NA objective). In the latter case, image acquisitions were performed using a Scion CFW1312M CCD camera driven from an Apple Macintosh G4 computer and homemade software. Data were quantified using the ImageJ software.

### Statistical analysis

Quantitative data are the means ± s.e.m. of at least four independent experiments. Paclitaxel cytotoxicity measurements were compared using multiple covariance analysis on StatView 5 software (SAS institute). The following symbols were used: *p ≤ 0.05, **p ≤ 0.01 and *** *p* ≤ 0.001.

## Supplementary information

Supplementary text

Figure S1

Figure S2

Figure S3

Figure S4

Figure S5

## References

[1] Murray S, Briasoulis E, Linardou H, Bafaloukos D, Papadimitriou C (2012). Taxane resistance in breast cancer: mechanisms, predictive biomarkers and circumvention strategies. Cancer Treat. Rev..

[2] Froidevaux-Klipfel L (2011). Modulation of septin and molecular motor recruitment in the microtubule environment of the Paclitaxel-resistant human breast cancer cell line MDA-MB-231. Proteomics.

[3] Froidevaux-Klipfel L (2015). Septin cooperation with tubulin polyglutamylation contributes to cancer cell adaptation to taxanes. Oncotarget.

[4] Fung KYY, Dai L, Trimble WS (2014). Cell and molecular biology of septins. Int. Rev. Cell. Mol. Biol..

[5] Montagna C, Bejerano-Sagie M, Zechmeister JR (2015). Mammalian septins in health and disease. Res. Rep. Biochem..

[6] Peterson EA, Petty EM (2010). Conquering the complex world of human septins: implications for health and disease. Clin. Genet..

[7] Kinoshita M, Field CM, Coughlin ML, Straight AF, Mitchison TJ (2002). Self- and actin-templated assembly of Mammalian septins. Dev. Cell.

[8] Sellin ME, Sandblad L, Stenmark S, Gullberg M (2011). Deciphering the rules governing assembly order of mammalian septin complexes. Mol. Biol. Cell.

[9] Kalikin LM, Sims HL, Petty EM (2000). Genomic and expression analyses of alternatively spliced transcripts of the MLL septin-like fusion gene (MSF) that map to a 17q25 region of loss in breast and ovarian tumors. Genomics.

[10] Scott M, McCluggage WG, Hillan KJ, Hall PA, Russell SE (2006). Altered patterns of transcription of the septin gene, SEPT9, in ovarian tumorigenesis. Int. J. Cancer.

[11] Stanbery L (2010). High SEPT9_v1 expression is associated with poor clinical outcomes in head and neck squamous cell carcinoma. Transl. Oncol..

[12] Gonzalez ME (2007). High SEPT9_v1 expression in human breast cancer cells is associated with oncogenic phenotypes. Cancer Res..

[13] Connolly D (2011). Septin 9 isoform expression, localization and epigenetic changes during human and mouse breast cancer progression. Breast Cancer Res..

[14] Chacko AD (2012). Expression of the SEPT9_i4 isoform confers resistance to microtubule-interacting drugs. Cell. Oncol..

[15] Bertin A (2010). Phosphatidylinositol-4,5-bisphosphate promotes budding yeast septin filament assembly and organization. J. Mol. Biol..

[16] Kinoshita M (1997). Nedd5, a mammalian septin, is a novel cytoskeletal component interacting with actin-based structures. Genes Dev..

[17] Silverman-Gavrila RV, Silverman-Gavrila LB (2008). Septins: new microtubule interacting partners. Sci. World J..

[18] Spiliotis ET (2010). Regulation of microtubule organization and functions by septin GTPases. Cytoskeleton.

[19] Wloga D, Gaertig J (2010). Post-translational modifications of microtubules. J. Cell Sci..

[20] Hallak ME, Rodriguez JA, Barra HS, Caputto R (1977). Release of tyrosine from tyrosinated tubulin. Some common factors that affect this process and the assembly of tubulin. FEBS Lett..

[21] Aillaud C (2017). Vasohibins/SVBP are tubulin carboxypeptidases (TCPs) that regulate neuron differentiation. Science.

[22] Ersfeld K (1993). Characterization of the tubulin-tyrosine ligase. J. Cell Biol..

[23] Banerjee A (2002). Increased levels of tyrosinated alpha-, beta(III)-, and beta(IV)-tubulin isotypes in paclitaxel-resistant MCF-7 breast cancer cells. Biochem. Biophys. Res. Commun..

[24] Bonnet C (2001). Differential binding regulation of microtubule-associated proteins MAP1A, MAP1B, and MAP2 by tubulin polyglutamylation. J. Biol. Chem..

[25] Lacroix B (2010). Tubulin polyglutamylation stimulates spastin-mediated microtubule severing. J. Cell. Biol..

[26] Magiera MM, Janke C (2014). Posttranslational modifications of tubulin. Curr. Biol..

[27] Song Y, Brady ST (2015). Post-translational modifications of tubulin: pathways to functional diversity of microtubules. Trends Cell Biol..

[28] McGrogan, B. T., Gilmartin, B., Carney, D. N. & McCann, A. Taxanes, microtubules and chemoresistant breast cancer. *Biochim. Biophys. Acta* **1785**, 96–132 (2008). 10.1016/j.bbcan.2007.10.004 18068131

[29] Peris L (2006). Tubulin tyrosination is a major factor affecting the recruitment of CAP-Gly proteins at microtubule plus ends. J. Cell Biol..

[30] Peris L (2009). Motor-dependent microtubule disassembly driven by tubulin tyrosination. J. Cell Biol..

[31] Bai X (2013). Novel septin 9 repeat motifs altered in neuralgic amyotrophy bind and bundle microtubules. J. Cell Biol..

[32] Sellin ME, Stenmark S, Gullberg M (2014). Cell type-specific expression of SEPT3-homology subgroup members controls the subunit number of heteromeric septin complexes. Mol. Biol. Cell.

[33] Smith C (2015). Septin 9 exhibits polymorphic binding to F-actin and inhibits myosin and cofilin activity. J. Mol. Biol..

[34] Janke C, Rogowski K, van Dijk J (2008). Polyglutamylation: a fine-regulator of protein function? ‘Protein Modifications: beyond the usual suspects’ review series. EMBO Rep..

[35] Rogowski K (2010). A family of protein-deglutamylating enzymes associated with neurodegeneration. Cell.

[36] Berezniuk I (2012). Cytosolic carboxypeptidase 1 is involved in processing α- and β-tubulin. J. Biol. Chem..

[37] Sangrajrang S (1998). Association of estramustine resistance in human prostatic carcinoma cells with modified patterns of tubulin expression. Biochem. Pharmacol..

[38] Mostowy S, Cossart P (2012). Septins: the fourth component of the cytoskeleton. Nat. Rev. Mol. Cell Biol..

[39] Bridges AA, Gladfelter AS (2015). Septin form and function at the cell cortex. J. Biol. Chem..

[40] Oegema K, Savoian MS, Mitchison TJ, Field CM (2000). Functional analysis of a human homologue of the Drosophila actin binding protein anillin suggests a role in cytokinesis. J. Cell Biol..

[41] Joo E, Surka MC, Trimble WS (2007). Mammalian SEPT2 is required for scaffolding nonmuscle myosin II and its kinases. Dev. Cell.

[42] Surka MC, Tsang CW, Trimble WS (2002). The mammalian septin MSF localizes with microtubules and is required for completion of cytokinesis. Mol. Biol. Cell.

[43] Nagata K (2003). Filament formation of MSF-A, a mammalian septin, in human mammary epithelial cells depends on interactions with microtubules. J. Biol. Chem..

[44] Poüs C, Klipfel L, Baillet A (2016). Cancer-related functions and subcellular localizations of septins. Front. Cell Dev. Biol..

[45] Spiliotis ET (2018). Spatial effects - site-specific regulation of actin and microtubule organization by septin GTPases. J. Cell Sci..

[46] Chacko AD (2005). SEPT9_v4 expression induces morphological change, increased motility and disturbed polarity. J. Pathol..

[47] Dolat L (2014). Septins promote stress fiber-mediated maturation of focal adhesions and renal epithelial motility. J. Cell Biol..

[48] Kremer BE, Adang LA, Macara IG (2007). Septins regulate actin organization and cell-cycle arrest through nuclear accumulation of NCK mediated by SOCS7. Cell.

[49] Spiliotis ET, Gladfelter AS (2012). Spatial guidance of cell asymmetry: septin GTPases show the way. Traffic.

[50] Spiliotis ET, Hunt SJ, Hu Q, Kinoshita M, Nelson WJ (2008). Epithelial polarity requires septin coupling of vesicle transport to polyglutamylated microtubules. J. Cell Biol..

[51] Kremer BE, Haystead T, Macara IG (2005). Mammalian septins regulate microtubule stability through interaction with the microtubule-binding protein MAP4. Mol. Biol. Cell.

[52] Beise N, Trimble W (2011). Septins at a glance. J. Cell Sci..

[53] Liu M, Shen S, Chen F, Yu W, Yu L (2010). Linking the septin expression with carcinogenesis. Mol. Biol. Rep..

[54] Stanbery L, Petty EM (2012). Steps solidifying a role for SEPT9 in breast cancer suggest that greater strides are needed. Breast Cancer Res..

[55] Verdier-Pinard P (2017). Septin9_i2 is downregulated in tumors, impairs cancer cell migration and alters subnuclear actin filaments. Sci. Rep..

[56] Amir S, Golan M, Mabjeesh NJ (2010). Targeted knockdown of SEPT9_v1 inhibits tumor growth and angiogenesis of human prostate cancer cells concomitant with disruption of hypoxia-inducible factor-1 pathway. Mol. Cancer Res..

[57] Gilad R (2015). High SEPT9_i1 Protein expression is associated with high-grade prostate cancers. PLoS ONE.

[58] Amir S, Mabjeesh NJ (2007). SEPT9_V1 protein expression is associated with human cancer cell resistance to microtubule disrupting agents. Cancer Biol. Ther..

[59] Golan M, Mabjeesh NJ (2013). SEPT9_i1 is required for the association between HIF-1α and importin-α to promote efficient nuclear translocation. Cell Cycle.

[60] Tazat, K., Schindler, S., Deppeing, R. & Mabjeesh, N. J. Septin 9 isoform 1 (SEPT9_i1) specifically interacts with importin-a1 to drive hypoxia-inducible factor (HIF)-1a nuclear translocation. *Cytoskeleton*10.1002/cm.21450 (2018). 10.1002/cm.21450 29742803

[61] Gonzalez ME, Makarova O, Peterson EA, Privette LM, Petty EM (2009). Up-regulation of SEPT9_v1 stabilizes c-Jun-N-terminal kinase and contributes to itspro-proliferative activity in mammary epithelial cells. Cell Signal..

[62] Daire V (2009). Kinesin-1 regulates microtubule dynamics via a c-Jun N-terminal kinase-dependent mechanism. J. Biol. Chem..

[63] Nakos K, Rosenberg M, Spiliotis ET (2018). Regulation of microtubule plus end dynamics by septin 9. Cytoskeleton.

[64] Mavrakis M (2014). Septins promote F-actin ring formation by crosslinking actin filaments into curved bundles. Nat. Cell Biol..

[65] Farrugia AJ, Calvo F (2016). The Borg family of Cdc42 effector proteins Cdc42EP1-5. Biochem. Soc. Trans..

[66] Bai X, Karasmanis EP, Spiliotis ET (2016). Septin 9 interacts with kinesin KIF17 and interferes with the mechanism of NMDA receptor cargo binding and transport. Mol. Biol. Cell.

[67] Nölke T (2016). Septins guide microtubule protrusions induced by actin-depolymerizing toxins like *Clostridium difficile* transferase (CDT). Proc. Natl Acad. Sci. USA.

