# Supplementary material for: Septin filament coalignment with microtubules depends on SEPT9_i1 and tubulin polyglutamylation, and is an early feature of acquired cell resistance to paclitaxel
Source: Cell Death Dis. 2019 Jan 22;10(2):54. doi: 10.1038/s41419-019-1318-6 (PMC6342940; doi:10.1038/s41419-019-1318-6)
Supplement: Supplementary file 1 — Supplementary text [file 41419_2019_1318_MOESM1_ESM.docx]

**Supplementary Information**

**Materials and Methods**

**Subcellular fractionation experiments**

MT-containing fractions were prepared as described earlier^1^ with minor modifications. Briefly, the cytosol was first extracted in a warm PEM (80mM PIPES, 2mM EGTA, 1mM MgCl_2_, pH 6.9) buffer containing 0.05% Triton X-100 with a mixture of antiproteases and antiphosphatases for 3min at 37°C. After one rinse with warm PEM, a MT-enriched fraction was recovered using ice-cold PM (80mM PIPES, 1mM MgCl_2_, pH 6.9) buffer supplemented with 5mM CaCl_2_, antiproteases and antiphosphatases (1h on ice). After one rinse in PM buffer, the insoluble non-MT-containing fractions were collected by scraping cell ghosts in hot Laemmli’s sample buffer, boiled (5min) and centrifuged. Supernatants and cell fractions were stored at -80°C prior to analysis.

**Figure legends**

*Figure S1 illustrates that all our plasmids are properly expressed in transfected cells. We indeed ensured that the transfection efficacy of each cell type was roughly similar whatever the set of plasmid combinations we used. We also checked by immunoblot that these combinations led to expected enzyme expression by detecting the appropriate tags or by following tubulin polyglutamylation (polyE).*

**Figure S1: Assessment of transfection efficacy and of polyglutamylation enzyme and/or of septin overexpression.**

(A) Transfection efficacy (expressed as percentages) was calculated for the four major combinations of plasmids used in the study in the different transfected cell types. n = number of cells analyzed. Data are the mean ± s.e.m. of at least three independent measurements (B-D) MDA-MB 231 Ts sensitive cells were transfected with the indicated cDNA mixtures. (B) Transfections were performed using 0.15 (no mark) or 0.25 µg (*) of each plasmid per well of 6-well plates. The expression of septins 2, 6 and 7 yielded 3 bands that can be visualized in two different tracks on the right. (C) The overexpression of both untagged TTLL5 and TTLL11 to allow long-chain tubulin polyglutamylation is evidenced by the polyE antibody and/or by the expression of CFP, which is coexpressed from the respective TTLL plasmids^2^. The overexpression of these polyglutamylation enzymes does not affect the parallel overexpression of septins, which is followed by revealing SEPT9, using either the anti-SEPT9 antibody (C) or the V5 tag, which is only present in the SEPT9_i1 construct (D).

*Figure S2 further checks the specificity of the effect of septin and polyglutamylase transfection on cell resistance induced by paclitaxel.* *We ascertained that, upon the reduction of tubulin polyglutamylation and of septin content, such paclitaxel treatments alone did not cause resistance.*

**Figure S2: Paclitaxel stimulation induces neither resistance nor the accumulation of CLIP-170 or MCAK when septin expression or the level of tubulin polyglutamylation is reduced.**

(A) MTT assays were conducted in paclitaxel-sensitive (Ts) MDA-MB 231 cells after knocking down SEPT2, SEPT9 or TTLL5 by RNAi and exposure to various concentrations of paclitaxel. Each bar is the mean ± s.e.m of at least 3 independent experiments. (B) Immunoblot analysis of the amount of CLIP-170 and MCAK after knocking down SEPT2, SEPT9 or TTLL5 by RNAi and exposure to 15nM paclitaxel for 24h. GT335 is a commercial antibody directed against polyglutamylated tubulin (Sigma).

*Figure S3 illustrates that cell death is reduced when a combination of septins and polyglutamylases is overexpressed, corroborating the MTT assay. Similar behavior of HuH7 cells at low paclitaxel concentration was also revealed, like in MTT assays (Fig. 3C).*

**Figure S3: Analysis of the release of histone-associated DNA fragments in the cytoplasm as a function of increasing concentrations of paclitaxel in transfected RPE-1, HeLa, CHO and HuH7 cells**

Cells were subjected to the overexpression of the indicated plasmids and cell death was quantified 24h after exposure to paclitaxel by measuring the cytoplasmic release of histone-associated DNA fragments relative to the values without paclitaxel. Each point is the mean ± s.e.m. of at least 3 independent experiments. * p ≤ 0.05, ** p ≤ 0.01

*Figure S4 shows the biochemical analysis of the subcellular relocalization of septin filaments (illustrated by SEPT9) towards the MT cytoskeleton following paclitaxel stimulation. With the exception of MDA-MB 231 Ts and HuH7 cells, which display a partial phenotype, all the other cell lines exhibit a dramatic relocalization of SEPT9 to MTs.*

**Figure S4: Biochemical analysis of SEPT9 compartmentalization between the insoluble non-MT-containing fractions (non-MT) and microtubule-enriched (MT) fractions before or after 24h paclitaxel treatment.**

After subcellular fractionation, SEPT9 repartition was analyzed by immunoblot before or after 24h paclitaxel treatment. The paclitaxel concentration used corresponds about to 3 times that of the IC_50_ of the cell line: MDA-MB 231 Ts, RPE-1, CHO and HuH7: 15nM; HeLa and HHL16: 5nM.

*Figure S5 shows the subcellular relocalization of septin filaments (illustrated by SEPT9 labelling) towards the MT cytoskeleton following acute paclitaxel treatment. Septin filaments are mainly associated with actin fibers in untreated cells while they co-align with MTs following paclitaxel treatment. It is to note that relocalization is incomplete in HuH7 cells, which is in agreement with the biochemical data.*

**Figure S5: Subcellular localization of septin filaments in HeLa and HuH7 cell lines in response to acute paclitaxel treatment.**

The overlay images show that septin filaments colocalize with actin stress fibers in both cell lines prior to exposure to paclitaxel, and co-align partially (HuH7) or more completely (HeLa) with MTs after 24h paclitaxel treatment. Endogenous SEPT9 labelling was used to detect endogenous septin filaments. The images shown are representative of at least 3 independent experiments. The white and cyan arrows show the partial co-alignment of septins with MTs and actin fibers, respectively. Scale bars = 10 µm.

**References**

1. Phung-Koskas, T. et al. STAT5B-mediated growth hormone signaling is organized by highly

dynamic microtubules in hepatic cells. *J. Biol. Chem.* **280,** 1123-1131 (2005).

2. Lacroix, B. et al. Tubulin polyglutamylation stimulates spastin-mediated microtubule severing. *J. Cell Biol.* **189,** 945-954 (2010).
