# Supplementary figures and images for: Septin filament coalignment with microtubules depends on SEPT9_i1 and tubulin polyglutamylation, and is an early feature of acquired cell resistance to paclitaxel
Source: Cell Death Dis. 2019 Jan 22;10(2):54. doi: 10.1038/s41419-019-1318-6 (PMC6342940; doi:10.1038/s41419-019-1318-6)

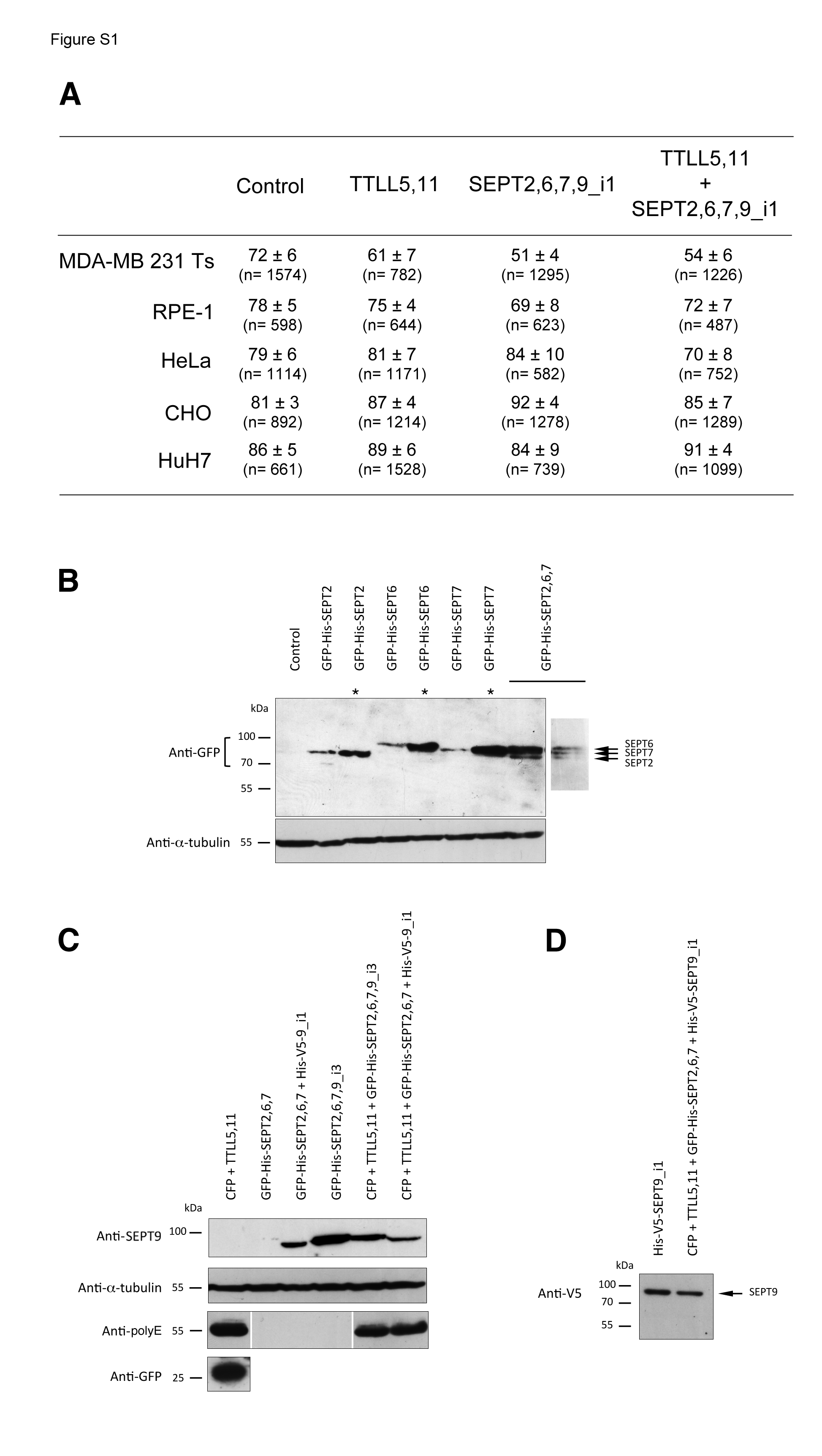

Supplement: Supplementary file 2 — Figure S1 [file 41419_2019_1318_MOESM2_ESM.jpg]

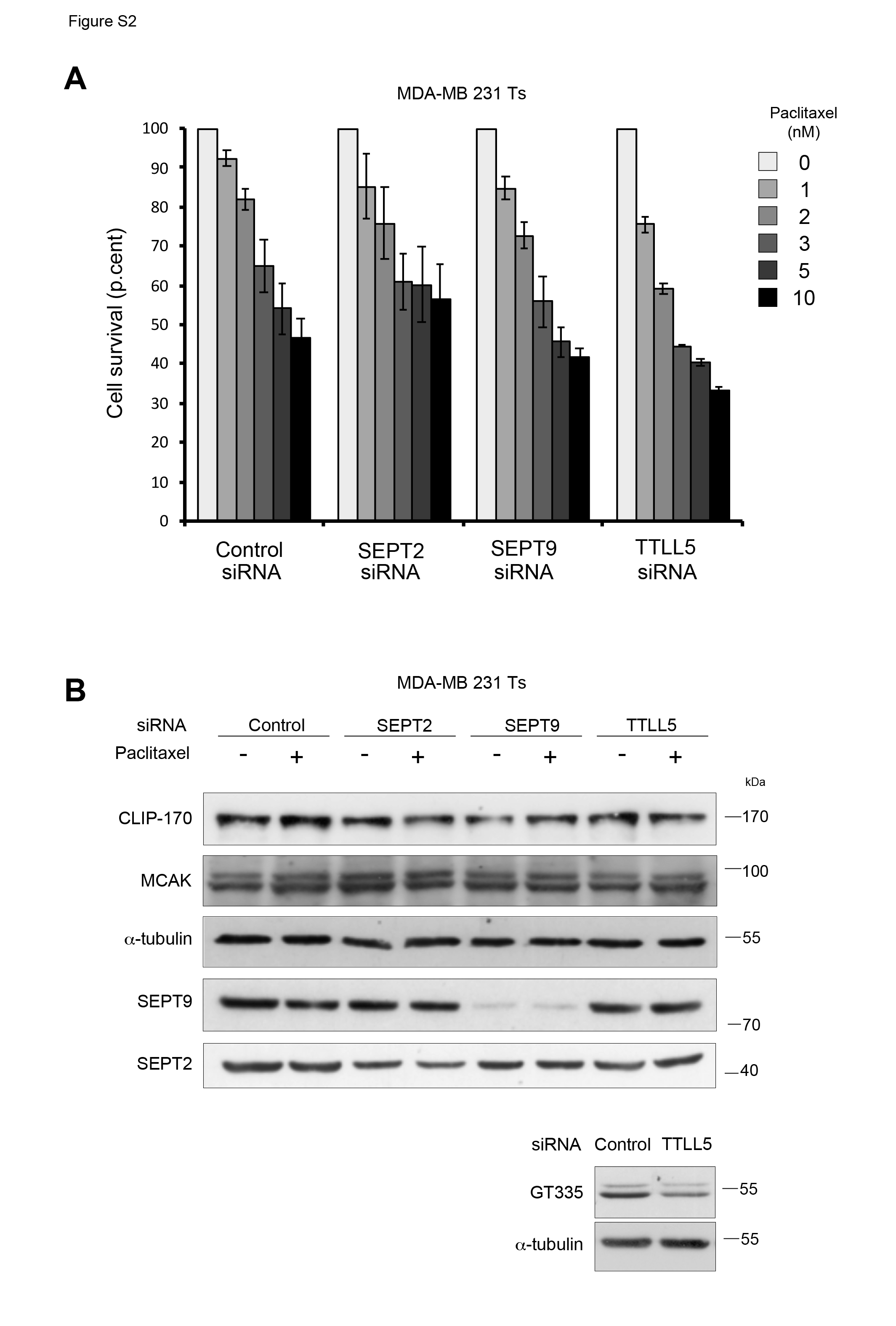

Supplement: Supplementary file 3 — Figure S2 [file 41419_2019_1318_MOESM3_ESM.jpg]

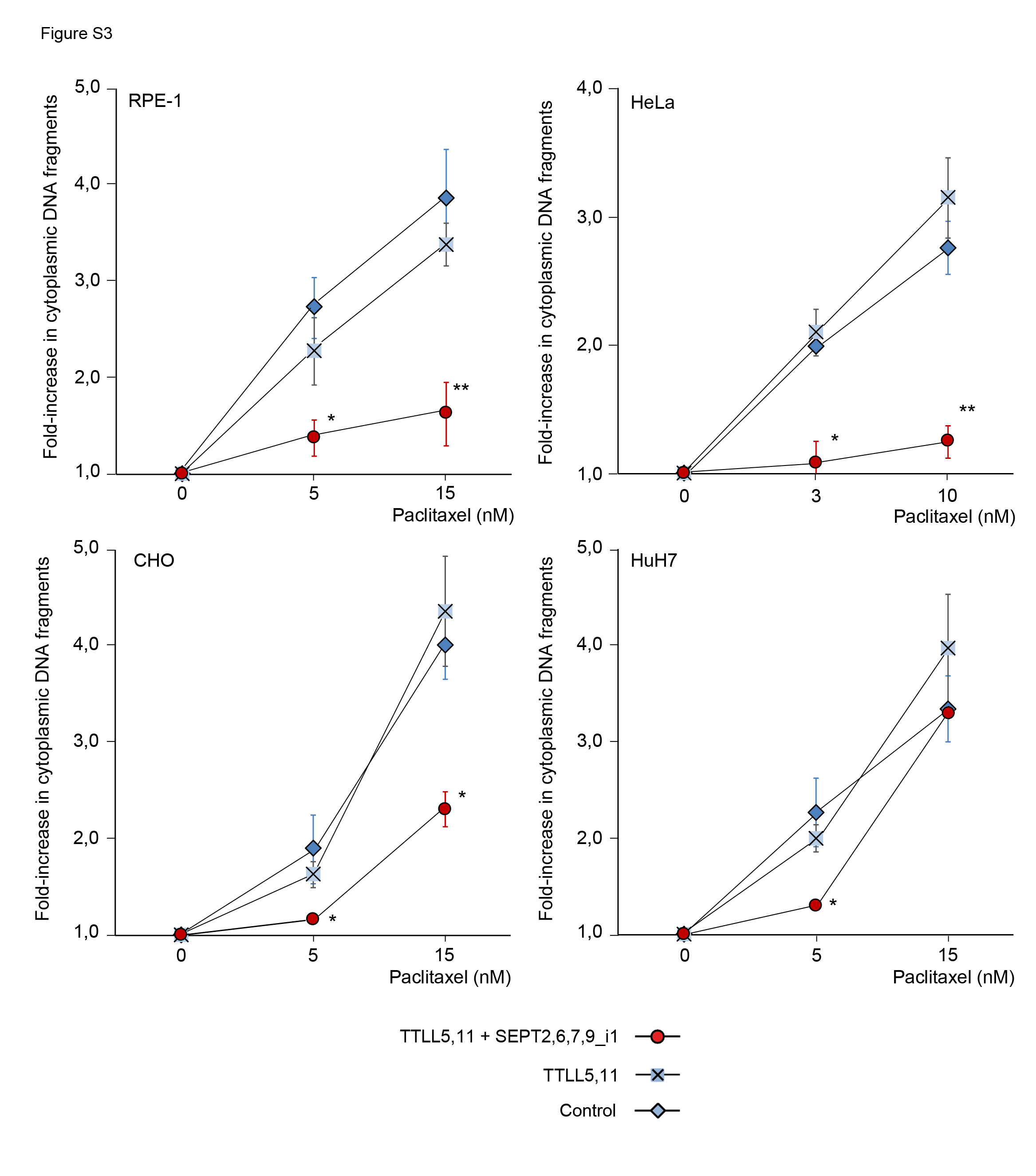

Supplement: Supplementary file 4 — Figure S3 [file 41419_2019_1318_MOESM4_ESM.jpg]

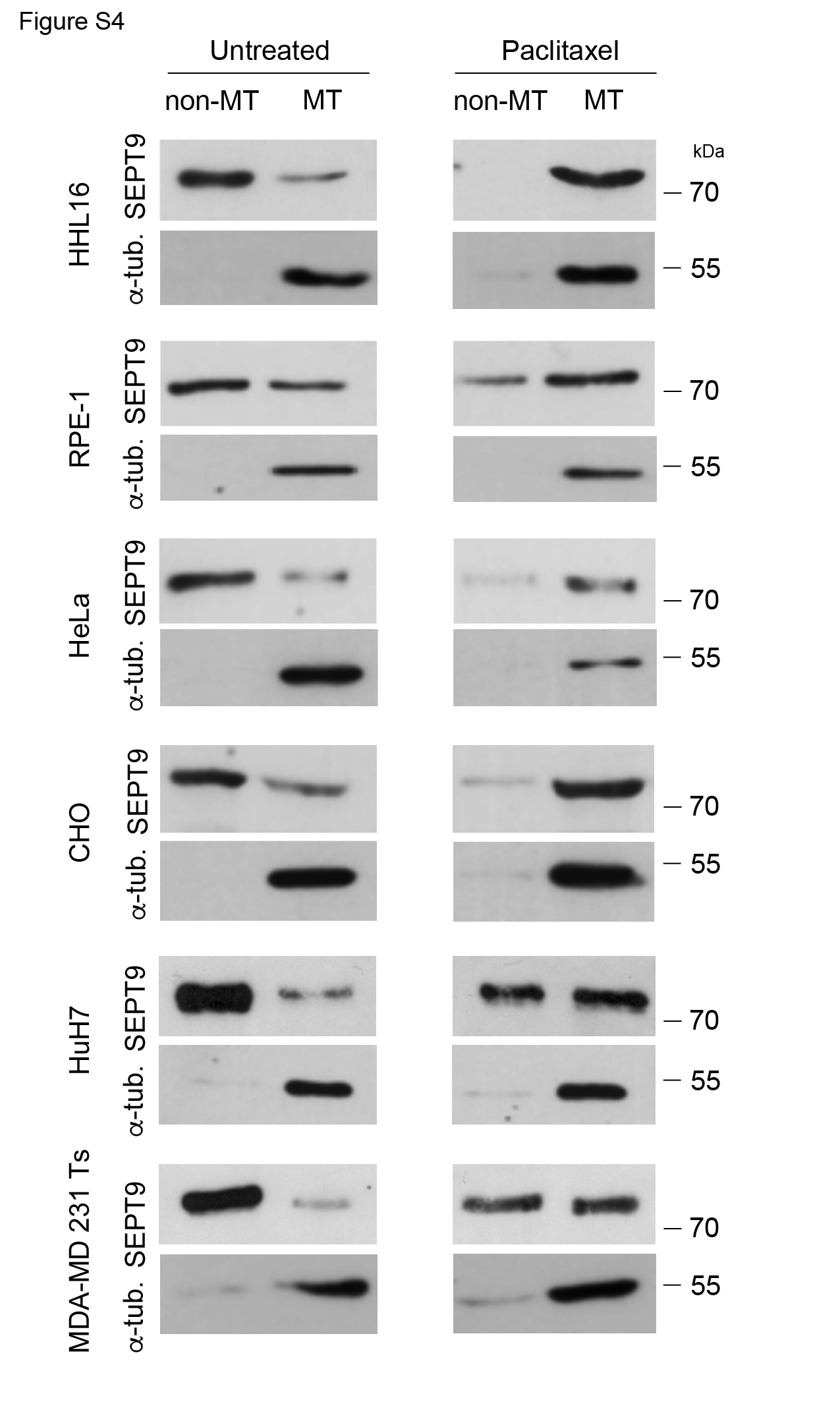

Supplement: Supplementary file 5 — Figure S4 [file 41419_2019_1318_MOESM5_ESM.jpg]

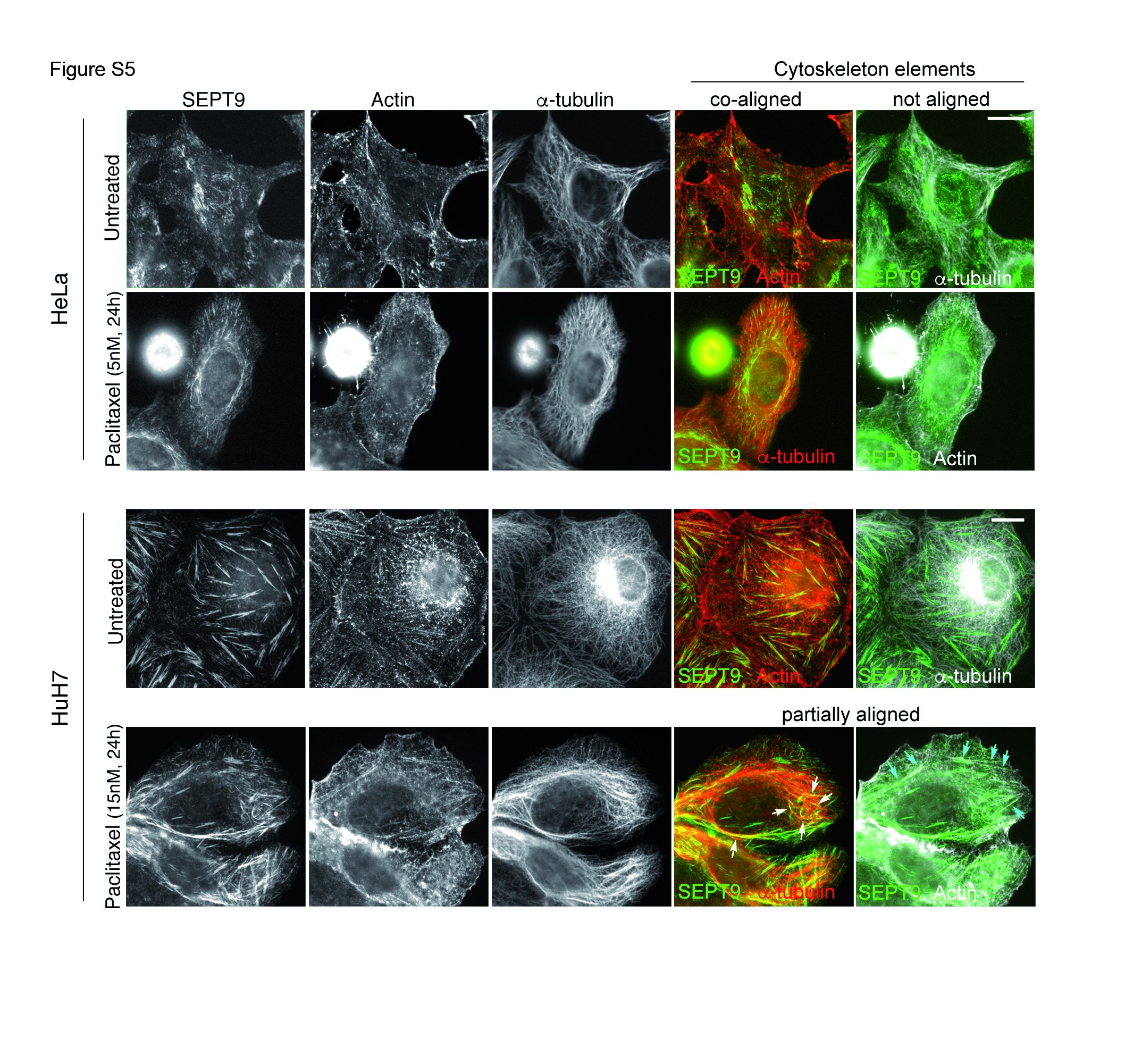

Supplement: Supplementary file 6 — Figure S5 [file 41419_2019_1318_MOESM6_ESM.jpg]
